# A model-guided holistic review of exploiting natural variation of photosynthesis traits in crop improvement

**DOI:** 10.1093/jxb/erac109

**Published:** 2022-03-22

**Authors:** Xinyou Yin, Junfei Gu, Michael Dingkuhn, Paul C Struik

**Affiliations:** 1 Centre for Crop Systems Analysis, Wageningen University & Research, PO Box 430, 6700 AK Wageningen, The Netherlands; 2 College of Agriculture, Yangzhou University, 48 Wenhui East Road, Yangzhou, Jiangsu 225009, China; 3 CIRAD, UMR 108 AGAP, F-34398 Montpellier, France; 4 University of Cambridge, UK

**Keywords:** Canopy traits, crop model, electron transport, QTL, source–sink relationships, trait synergy, yield improvement

## Abstract

Breeding for improved leaf photosynthesis is considered as a viable approach to increase crop yield. Whether it should be improved in combination with other traits has not been assessed critically. Based on the quantitative crop model GECROS that interconnects various traits to crop productivity, we review natural variation in relevant traits, from biochemical aspects of leaf photosynthesis to morpho-physiological crop characteristics. While large phenotypic variations (sometimes >2-fold) for leaf photosynthesis and its underlying biochemical parameters were reported, few quantitative trait loci (QTL) were identified, accounting for a small percentage of phenotypic variation. More QTL were reported for sink size (that feeds back on photosynthesis) or morpho-physiological traits (that affect canopy productivity and duration), together explaining a much greater percentage of their phenotypic variation. Traits for both photosynthetic rate and sustaining it during grain filling were strongly related to nitrogen-related traits. Much of the molecular basis of known photosynthesis QTL thus resides in genes controlling photosynthesis indirectly. Simulation using GECROS demonstrated the overwhelming importance of electron transport parameters, compared with the maximum Rubisco activity that largely determines the commonly studied light-saturated photosynthetic rate. Exploiting photosynthetic natural variation might significantly improve crop yield if nitrogen uptake, sink capacity, and other morpho-physiological traits are co-selected synergistically.

## Teaser

Exploiting photosynthetic natural variation can significantly improve yield if electron transport, nitrogen uptake, and morpho-physiological components are improved synergistically.

## Introduction

Crop yield needs improving in the face of growing populations, accelerating climate change, and diminishing land resources available for crop production. Photosynthesis scholars argue that this improvement most probably has to come from an enhanced photosynthesis (e.g. Long *et al.*, 2015; Ort *et al.*, 2015; Furbank *et al.*, 2020; Walter and Kromdijk, 2022).

Photosynthesis can be improved via a synthetic biology approach through genetic modification. For example, introducing multigenic C_4_ photosynthesis into C_3_ crops (von Caemmerer *et al.*, 2012) was predicted to increase yield significantly (Yin and Struik, 2017), but it is a long-shot challenge that will take many years to accomplish (Long *et al.*, 2015). Genetic modifications for simpler processes, such as accelerating recovery from photoprotection under fluctuating light (Kromdijk *et al.*, 2016) or bypassing photorespiration (South *et al.*, 2019), have resulted in more productive model plants under field conditions. However, inserting such modifications into major crops remains a challenge. Another approach is to exploit natural variation in photosynthesis among and within species (Flood *et al.*, 2011). Within species, natural variation in leaf photosynthesis has been reported for major crops (e.g. Gu *et al.*, 2012a; Driever *et al.*, 2014; Meena *et al.*, 2021). Exploiting such natural variation via conventional breeding is most likely to be the best short-term option.

While past yield improvements rarely came from increased photosynthesis and genotypic yields rarely correlate with leaf photosynthetic rates (e.g. Driever *et al.*, 2014; Gu *et al.*, 2014b), increased photosynthesis generally increases yield, for example in FACE (free-air CO_2_ enrichment) trials (e.g. Lv *et al.*, 2020). Increased photosynthetic rates, if achievable beyond those attained in modern cultivars, would thus be a promising objective. However, limited success has been achieved from various efforts in exploiting natural variation of photosynthetic traits to improve crops (e.g. Flexas, 2016), probably because natural variation in photosynthesis above the levels of present standard cultivars may be small. In addition, crop physiologists (e.g. Sinclair *et al.*, 2019; Araus *et al.*, 2021) argue that yield is a very complex trait, depending little on leaf photosynthesis but rather on many other physiological processes. This probably means that breeders should exploit variations in multiple traits and aggregate them synergistically in order to improve crop yield.

**Fig. 1.**
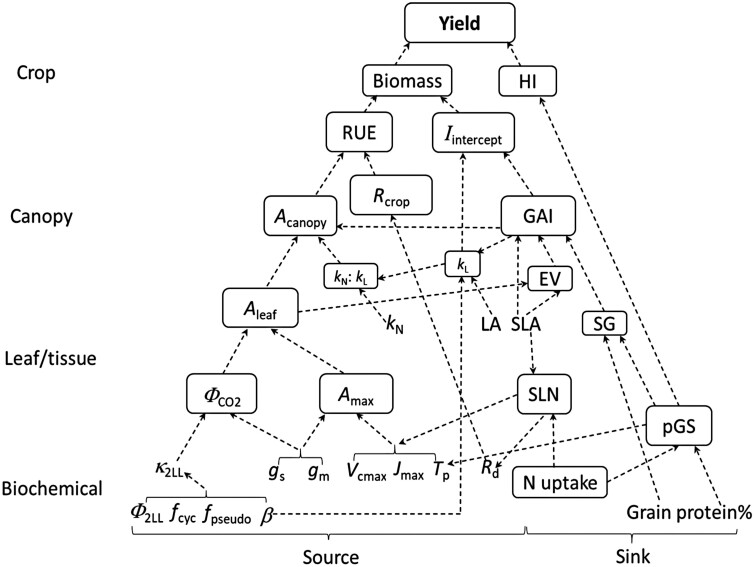
A simplified qualitative scheme of the quantitative crop model GECROS connecting hierarchical scales from biochemical parameters to crop yield, and covering both photosynthetic (source) and morpho-physiological (sink) traits. Items in rectangles are traits quantified in the model along the hierarchical scales, while those without rectangles are model parameters. Abbreviations and symbols: *A*_canopy_, canopy photosynthesis rate; *A*_leaf_, leaf photosynthesis rate; *A*_max_, maximum rate of light-saturated *A*_leaf_; EV, early vigour; *f*_cyc_, fraction for cyclic electron transport; *f*_pseudo_, fraction for pseudocyclic electron transport; GAI, green surface area index; *g*_m_, mesophyll conductance; *g*_s_, stomatal conductance; HI, harvest index; *I*_intercept_, photosynthetically active radiation intercepted by canopy; *J*_max_, maximum capacity of light-saturated linear electron transport; *k*_L_, light extinction coefficient in canopy; *k*_N_, leaf nitrogen extinction coefficient in canopy; LA, leaf angle; pGS, potential grain size; *R*_d_, leaf day respiration; *R*_crop_, crop respiration; RUE, radiation use efficiency; SG: stay-green; SLA, specific leaf area; SLN, specific leaf nitrogen content; *T*_p_, rate of triose phosphate utilization; *V*_cmax_, maximum carboxylation capacity of Rubisco; β, absorptance by leaf photosynthetic pigments; Φ_2LL_, quantum efficiency of electron transport of PSII under limiting light; Φ_CO2_, quantum efficiency of CO_2_ assimilation under limiting light; κ_2LL_, efficiency of converting incident light into linear electron transport under limiting light conditions. Further details of the scheme are described in Box 1 and in the main text.

In this review, we first describe a whole-plant physiology framework (Box 1), based on a quantitative crop model with parameters that capture multiple traits underlying yield hierarchy from photosynthetic biochemistry to leaf, to canopy, to crop scales (Fig. 1). Based on this framework, we review the extent of natural variation in relevant parameters along the yield hierarchy. We further review quantitative trait loci (QTL) for some of these parameters. We then use the same crop model to assess potential benefits from pyramiding favourable traits, and to identify most important parameters for improving yield beyond that of best-performing cultivars. We focus on C_3_ crops, given their economic importance and their greater improvement potential than C_4_ crops (Yin and Struik, 2015).

**Box 1. A general whole-plant physiology framework based on the crop model GECROS**

Crop yield depends on intercepted light or radiation (*I*_intercept_), radiation use efficiency (RUE) for conversion of *I*_intercept_ into biomass, and harvest index (HI), the fraction of biomass constituting the harvestable product. Yield improvement from the 1960s Green Revolution for major food crops resulted mainly from increased HI via dwarfing genes (Miflin, 2000; Hedden, 2003), although increased *I*_intercept_ and/or RUE also contributed in some cases (Sadras *et al.*, 2012; Koester *et al.*, 2014). As HI has reached a plateau, further yield improvement will require improving either *I*_intercept_ or RUE (e.g. Long *et al.*, 2015; Furbank *et al.*, 2020). In order to identify the components that can be exploited to improve yield, we describe biochemical and morpho-physiological components affecting *I*_intercept_, RUE, and HI (Fig. 1), according to the principles as captured in the crop model GECROS (Yin and Struik, 2017).

During the growing season, *I*_intercept_ is set by the green surface area duration [integrating the green surface area index (GAI) and how long it is sustained] and light extinction coefficient (*k*_L_) of the canopy. Early vigour promotes early canopy closure, and stay-green traits extend terminal GAI duration. For a given leaf mass, a high specific leaf area (SLA) enables rapid leaf expansion and increase of *I*_intercept_ (Dingkuhn *et al.*, 2001).

RUE depends on canopy photosynthesis (*A*_canopy_), the photosynthates lost by crop respiration (*R*_crop_), and the conversion efficiency of net photosynthates into biomass. The latter efficiency (Penning de Vries *et al.*, 1989) and the *R*_crop_ versus *A*_canopy_ ratio (Amthor, 2010) are conservative for a given species under favourable conditions. Photosynthetic competence of individual leaves affects *A*_canopy_; however, for given photosynthetic resources (especially nitrogen), their vertical distribution among canopy strata is also crucial. This distribution is described by the nitrogen extinction coefficient (*k*_N_). A canopy with similar *k*_N_ and *k*_L_ achieves a high *A*_canopy_ (Goudriaan, 1995; Sands, 1995).

Leaf photosynthetic competence (*A*_leaf_) depends on light-saturated photosynthetic capacity (*A*_max_) and the initial light–response slope of CO_2_ assimilation (syn. quantum yield, Φ_CO2_). The *A*_max_ mainly depends on either the maximum rate of linear electron transport (*J*_max_) or metabolic capacity parameters such as Rubisco activity (*V*_cmax_) and the capacity for triose phosphate utilization (*T*_p_) (see Equation 1). In theory, Φ_CO2_ depends primarily on the photochemical efficiency of PSII electron transport and whether there are cyclic and pseudo-cyclic pathways that drain electrons from CO_2_ assimilation (Yin *et al.*, 2006; see Equation 2A). As CO_2_ and O_2_ compete for active catalytic sites of Rubisco, the relative partial pressure of CO_2_ versus O_2_ at Rubisco-carboxylating sites will affect the amount of photorespiration. Therefore, parameters governing CO_2_ diffusion, including stomatal conductance (*g*_s_) and mesophyll conductance (*g*_m_) (see Equation 3), affect both *A*_max_ and Φ_CO2_.

Some of these parameters act at the canopy level while others act at the (sub)-foliar level. Some are morpho-physiological and others are biochemical. However, they are not independent. For example, SLA is a morphological parameter but influences biochemical parameters *J*_max_, *V*_cmax_, and *T*_p_ as they are commonly expressed per leaf area. A smaller SLA (thicker leaves) results in higher aerial nitrogen content, and higher values of *A*_max_ (Boote and Tollenaar, 1994) and underlying biochemical components *J*_max_, *V*_cmax_, and *T*_p_. In addition, while *T*_p_ is a biochemical parameter reflecting the local sink size for sucrose and starch synthesis in the leaf (Sharkey, 1985), recent evidence suggests that *T*_p_ is regulated by whole-plant source–sink relations (Fabre *et al*., 2019, 2020).

A greater potential sink demand for photosynthates via larger panicles can increase HI, yet this may impede ‘stay-green’ traits as grain growth requires nitrogen (Sinclair and de Wit, 1976) and most grain nitrogen comes from remobilization from vegetative organs, particularly leaves. Leaves are source organs, but also sink organs while growing. Increasing photosynthesis has a feedforward effect on leaf production and tillering/branching, and thus early vigour, enabling early canopy closure. However, leaf and tiller production as a sink may also feed back on leaf photosynthesis by removing sink limitation set by *T*_p_. This has been evidenced by genotypes with superior sink capacity responding more strongly to CO_2_ enrichment (Hasegawa *et al.*, 2013; Dingkuhn *et al.*, 2020; Yiotis *et al.*, 2021).

In short, exploiting natural variation of photosynthesis to increase crop yield potential is not a matter of increasing photosynthesis alone, but should be approached from a whole-plant perspective to exploit synergisms among multiple traits.

## Natural variation of photosynthetic parameters

### *A*_max_ and its underlying biochemical parameters

According to the (extended) model of Farquhar *et al.* (1980), light-saturated photosynthesis capacity (*A*_max_) can be expressed as:


$$A_{\text{max}}=\{\begin{array}{cc} \frac{\left(C_{\text{c}}-\text{ ​​}\Gamma \text{​​}{\;}_{*}\right)V_{\text{cmax}}}{C_{\text{c}}+K_{\text{mC}}\left(1+O/K_{\text{mO}}\right)}-R_{\text{d}} & \text{ifRubiscoactivitylimits}\\ \frac{\left(C_{\text{c}}-\text{ ​​}\Gamma \text{​​ *}\right)J_{\text{max}}}{4\left(C_{\text{c}}+2\text{ ​​}\Gamma \text{​​ *}\right)}-R_{\text{d}} & i\text{felectrontransportlimits}\\ \frac{\left(C_{\text{c}}-\text{ ​​}\Gamma \text{​​}{\;}_{*}\right)\left(3T_{p}\right)}{C_{\text{c}}-\left(1+4\text{ ​​}\alpha \text{​​}{\text{ S}}_{\text{*}}\right)\text{ ​​}\Gamma \text{​​ *}}-R_{\text{d}} & \text{ifTPUlimits} \end{array}$$ (1)

where *C*_c_ is the level of CO_2_ in the chloroplast, Γ_*_ is the CO_2_ compensation point in the absence of day respiration (*R*_d_), *K*_mC_ and *K*_mO_ are the Michaelis–Menten constants of Rubisco for CO_2_ and O_2_, respectively, *V*_cmax_ is the maximum carboxylation capacity of Rubisco, *J*_max_ is the maximum capacity of linear electron transport, *T*_p_ is the rate of triose phosphate utilization (TPU), and α_S_ is the fraction of the glycolate-carbon that does not return to chloroplasts but exits via the photorespiratory pathway (with 0≤α_S_ ≤0.75), assuming that serine is the major form of carbon that exits (see Busch *et al.*, 2018; Yin *et al.*, 2021). As *C*_c_ for a given ambient [CO_2_] depends on stomatal and mesophyll conductance as discussed later, and Γ_*_, *K*_mC_, and *K*_mO_ are Rubisco kinetic parameters that are very conserved within C_3_ species, *A*_max_ depends mainly on biochemical capacity parameters *V*_cmax_, *J*_max_, or *T*_p_.

A large variation in *A*_max_ has been reported for various crops. Ye *et al.* (2019) showed ~2-fold variation of *A*_max_ among 121 rice cultivars, ranging from 15.5 μmol m^−2^ s^−1^ to 32.6 μmol m^−2^ s^−1^, while Qu *et al.* (2017) showed *A*_max_ varying from 13.7 μmol m^−2^ s^−1^ to 28.2 μmol m^−2^ s^−1^ among 214 rice genotypes. A variation of *A*_max_ among 64 wheat cultivars was reported by Driever *et al.* (2014), ranging from 20.5 μmol m^−2^ s^−1^ to 31.5 μmol m^−2^ s^−1^. Sadras *et al.* (2012) showed a variation of *A*_max_ from 9.3 μmol m^−2^ s^−1^ to 19.6 μmol m^−2^ s^−1^ for 13 Australian wheat cultivars released between 1958 and 2007. Shrestha *et al.* (2018) showed an ~1.7-fold range in *A*_max_ among 20 chickpea genotypes. Jin *et al.* (2010) showed a 33% increase in *A*_max_ for 45 soybean cultivars released in 1950 to 2006 in China, an increase of 0.067 μmol m^−2^ s^−1^ year^–1^.

Where CO_2_ or light response curves are measured that allow the estimation of *V*_cmax_ and *J*_max_, the variation of *A*_max_ is often associated with *V*_cmax_ or *J*_max_, or both (Driever *et al.*, 2014; Jahan *et al.*, 2014; Carmo-Silva *et al.*, 2017; Silva-Perez et al., 2020; Acevedo-Siaca *et al.*, 2021). In general, *V*_cmax_ and *J*_max_ estimated across genotypes/accessions are highly correlated (Driever *et al.*, 2014; McAusland *et al.*, 2020; Mathan *et al.*, 2021). Carmo-Silva *et al.* (2017) and Acevedo-Siaca *et al.* (2021) showed that both parameters had high heritabilities.

*A*_max_, *V*_cmax_, and *J*_max_ are commonly expressed on a leaf area basis, and their genotypic variation may be related to leaf thickness or to nitrogen content (e.g. Mathan *et al.*, 2021; Fig. 1). An increase in *A*_max_ resulting from thicker leaves is not desirable (Austin, 1989) because of trade-offs with leaf expansion, and thus light interception, potentially causing a decrease in whole-plant photosynthesis (Boote and Tollenaar, 1994; Richards, 2000). Likewise, variation in *A*_max_ depends on leaf nitrogen, which probably results from differences in root uptake (Hikosaka, 2010). Thus, it is useful to correct *A*_max_ for the difference in specific leaf nitrogen (SLN, g N m^−2^) that captures both leaf thickness and leaf nitrogen concentration; that is, expressing leaf photosynthetic capacity as so-called photosynthetic nitrogen use efficiency (PNUE=*A*_max_/SLN). However, PNUE still varied by ~2-fold among rice genotypes, from 10.0 μmol (g N)^−1^ s^−1^ to 22.6 μmol (g N)^−1^ s^−1^ (Ye *et al.*, 2019). Silva-Perez *et al*. (2020) also showed significant genotypic variations in *V*_cmax_ or *J*_max_ to SLN ratios.

### Φ_CO2_ and its components

The initial slope of the photosynthetic light response curve, also called photosynthetic quantum efficiency (Φ_CO2_), is a composite parameter (Yin *et al.*, 2006):


$$\begin{array}{l} \Phi_{CO2}=\Phi_{2LL}\frac{1-f_{cyc}-f_{pseudo}}{\Phi_{2LL}/\Phi_{1LL}+(1-f_{cyc})}\;\frac{C_{c}-\Gamma_{*}}{4(C_{c}+2\Gamma_{*})} \end{array}$$ (2A)

where Φ_2LL_ is the photochemical efficiency of PSII under limiting light conditions, *f*_cyc_ and *f*_pseudo_ are the fractions for cyclic and pseudocyclic electron transport, and Φ_2LL_/Φ_1LL_ is the PSII to PSI photochemical efficiency ratio, which is presumably conserved (~0.85; see Yin *et al.*, 2021, and references therein). If photosynthetic quantum efficiency is expressed on the incident light basis (Φ_CO2,inc_), it can be set as: Φ_CO2,inc_=βΦ_CO2_, where β is absorptance by leaf photosynthetic pigments. As *f*_cyc_ and *f*_pseudo_ are hard to determine and may be conserved within C_3_ species under limiting light (Yin *et al.*, 2006), Φ_CO2,inc_ is commonly expressed as:


$$\begin{array}{l} {\;\Phi \;}_{CO2,inc}=\kappa_{2LL}\frac{C_{c}-{\;\Gamma }_{*}}{4\left(C_{c}+2{\;\Gamma }_{*}\right)} \end{array}$$ (2B)

where κ_2LL_, lumping β, Φ_2LL_, and the second term of Equation 2A, represents the efficiency of converting incident light into linear electron transport.

Values of Φ_CO2_ or Φ_CO2,inc_ are obtained from linear regression of photosynthetic light–response curves within a limiting light range, or from curvilinear regression of the light–response curves over a broader light range. Consequently, phenotyping Φ_CO2_ for a large number of genotypes is time consuming. Furthermore, Φ_CO2_ is very conserved (Austin, 1989), even across various C_3_ species (Björkman and Demmig, 1987), and measurement or curve-fitting errors can obscure differences among genotypes. For example, Carmo-Silva *et al.* (2017) detected little genotypic differences for wheat in Φ_CO2,inc_ estimated from curvilinear regression.

Instead of measuring Φ_CO2_, phenotyping *A* at a low light level (e.g. 100 μmol m^−2^ s^−1^) *A*_low_, or phenotyping the components of Φ_CO2_ may help find genetic differences in Φ_CO2_. Qu *et al.* (2017) phenotyped *A*_low_ for ~200 rice genotypes at two locations and found >2-fold genotypic differences at each location, although location effect and location×genotype interaction were more significant than genotype effect.

Of the components determining Φ_CO2_, only Φ_2LL_ (Equation 2A, B) can be relatively easily phenotyped from assessing chlorophyll fluorescence parameter *Fʹ*_v_/*Fʹ*_m_. A good proxy is the more widely available parameter *F*_v_/*F*_m_, the quantum efficiency of PSII photochemistry in dark-adapted leaves. Carmo-Silva *et al.* (2017) showed a significant correlation between *Fʹ*_v_/*Fʹ*_m_ and *F*_v_/*F*_m_, and both were highly heritable in 64 wheat cultivars. Similarly, Czyczyło-Mysza *et al.* (2013) reported small but significant differences in *F*_v_/*F*_m_ among 91 wheat genotypes. Qu *et al.* (2017) showed that *F*_v_/*F*_m_ positively correlated with *A*_low_ among 214 rice genotypes.

Leaf absorptance β is relevant for improving Φ_CO2,inc_. It depends on leaf chlorophyll content [CHL] which is easily assessed with a SPAD leaf greenness meter. [CHL] systematically increased with the year of release of 24 soybean cultivars (Koester *et al.*, 2016). The SPAD values varied significantly among genotypes in wheat (Giunta *et al.*, 2002; Sadras *et al.*, 2012; Czyczyło-Mysza *et al.*, 2013; Carmo-Silva *et al.*, 2017; Silva-Perez *et al*., 2020), rice (Qu *et al.*, 2017; Zhu *et al.*, 2020), and barley (Giunta *et al.*, 2002). However, while increasing β is beneficial for improving leaf-level Φ_CO2,inc_, in the canopy context an increased β leads to an increased light extinction coefficient (*k*_L_), decreasing the contribution of lower leaves to canopy photosynthesis (see a later section).

### CO_2_ diffusion parameters *g*_s_ and *g*_m_

Both *A*_max_ and Φ_CO2_ depend on the level of chloroplast CO_2_ (*C*_c_) (see Equations 1, 2A). CO_2_ molecules from the atmosphere have to cross resistance barriers to reach chloroplasts which can be expressed as:


$$\begin{array}{l} C_{c}=C_{i}-A/g_{m}=C_{a}-A\left(\frac{1}{g_{m}}+\frac{1}{g_{s}}\right) \end{array}$$ (3)

where *C*_a_ and *C*_i_ are the level of CO_2_ at the atmosphere and intercellular air spaces, respectively, and *g*_s_ and *g*_m_ are stomatal and mesophyll conductance, respectively (where 1/*g*_s_ also includes the boundary layer resistance).

*g*_s_ varies with environment. When assessed under given conditions, *g*_s_ for water vapour has been reported to vary: 0.14–1.16 mol m^−2^ s^−1^ for ~200 rice genotypes under saturating light and 0.17–0.26 mol m^−2^ s^−1^ under low-light conditions (Qu *et al.*, 2017), and 0.2–1.0 mol m^−2^ s^−1^ for various sets of wheat panels (Silva-Perez *et al*., 2020). Genotypic differences in *g*_s_ were also significant within samples of a smaller number of genotypes in rice (Jahn *et al.*, 2011), wheat (Jahan *et al.*, 2014), soybean (Koester *et al.*, 2016; Tomeo and Rosenthal, 2017), and chickpea (Shrestha *et al.*, 2018). The heritability of *g*_s_ was high (mostly >0.5) in wheat (Carmo-Silva *et al.*, 2017; Silva-Perez *et al*., 2020). Anatomical parameters (e.g. stomatal density, stomatal length, and stomatal width) underlying *g*_s_ also varied among genotypes in wheat (Sadras *et al.*, 2012; Ouyang *et al.*, 2017) and in rice (Ouyang *et al.*, 2017). Further information on natural variation of *g*_s_ can be found in recent reviews (Nunes-Nesi *et al.*, 2016; Faralli and Lawson, 2020), and many studies demonstrated a yield increase associated with increased *g*_s_ (Fischer *et al.*, 1998; Richards, 2000).

Compared with the information on *g*_s_ for large genetic panels, datasets for *g*_m_ are smaller, probably because *g*_m_ is difficult to measure. When estimated by the carbon isotope method, *g*_m_ varied from 0.05 mol m^−2^ s^−1^ bar^−1^ to 0.50 mol m^−2^ s^−1^ bar^−1^ in six barley genotypes (Barbour *et al.*, 2010), from 0.5 mol m^−2^ s^−1^ bar^−1^ to 1.0 mol m^−2^ s^−1^ bar^−1^ in 10 wheat genotypes (Jahan *et al.*, 2014), and from 0.29 mol m^−2^ s^−1^ bar^−1^ to 0.88 mol m^−2^ s^−1^ bar^−1^ among 20 chickpea genotypes (Shrestha *et al.*, 2018). Using the constant J method, Koester *et al.* (2016) showed that *g*_m_ in 24 soybean genotypes varied from 0.10 mol m^−2^ s^−1^ bar^−1^ to 0.26 mol m^−2^ s^−1^ bar^−1^, and the values were not related to the cultivars’ year of release. Using a chlorophyll fluorescence-based method, Tomeo and Rosenthal (2017) showed for 12 soybean cultivars that *g*_m_ varied by >2-fold, with 38% of this variation caused by genotype. Using a similar method, Ouyang *et al.* (2017) showed that *g*_m_ of upland rice genotypes was ~50% lower than that of lowland rice genotypes, and *g*_m_ of lowland rice was ~50% lower than that of wheat genotypes, confirming the high values of *g*_m_ reported by Jahan *et al.* (2014) for wheat. Ouyang *et al.* (2017) further showed that *S*_c_/*S*_m_ (ratio of the exposed surface area of chloroplasts to the exposed surface area of mesophyll cell walls) contributed most to variation in *g*_m_ among rice genotypes, whereas *T*_w_ (thickness of the mesophyll cell wall) was the main determinant of *g*_m_ in wheat. Scafaro *et al.* (2011) and Giuliani *et al.* (2013) reported that *T*_w_ in rice and its wild relatives was highly correlated with *g*_m_.

Increasing *g*_s_ can increase both leaf photosynthesis and transpiration, whereas *g*_m_ increases leaf photosynthesis but not transpiration. A high *g*_m_ or a high *g*_m_:*g*_s_ ratio can thus improve leaf-level transpiration efficiency (Flexas *et al.*, 2013). A good correlation of transpiration efficiency versus *g*_m_:*g*_s_ has been reported across 15 soybean cultivars (Bunce, 2016), nine rice and wheat genotypes (Ouyang *et al.*, 2017), and across 24 accessions of cultivated rice and its wild relatives (Giuliani *et al.*, 2013).

### Stimulating photosynthesis by increasing sink size

The effect of sink activity on photosynthesis has long been known (e.g. Paul *et al.*, 1992), and occurs at both leaf- and whole-plant scales. The sink limitation at leaf level for a short time scale is reflected by the ability to utilize triose phosphate, the product of carbon reduction in the Calvin–Benson cycle, for sucrose or starch synthesis (Sharkey 1985; see Equation 1). As the half-life time of the intermediates in the Calvin–Benson cycle is shorter than that in sucrose or starch synthesis, the limitation set by TPU can build up and disappear quickly. Therefore, the TPU limitation is not always observable as other components such as electron transport are regulated to counteract TPU limitation (e.g. Sharkey, 2019). At the whole-plant scale over a longer time span, the sink–source (im)balance often refers to whether available photosynthates satisfy or exceed the demand for growth of panicles, stems, roots, and leaves. It has been observed that larger sinks can stimulate photosynthesis of source organs (reviewed by Dingkuhn *et al.*, 2020). For example, Kikuchi *et al.* (2017) demonstrated that genotypic tillering capacity increased rice yield response to elevated [CO_2_]. Hasegawa *et al.* (2013) showed that among eight rice cultivars in Japan, those that responded most to the elevated [CO_2_] under FACE environments had larger reproductive sinks. The importance of sink traits was shown for a larger FACE dataset covering rice genotypes from Japan and China (Lv *et al.*, 2020). Gao *et al.* (2021) confirmed that a high sink/source ratio is necessary for higher photosynthesis and productivity under elevated [CO_2_].

There has been little communication between photosynthesis biologists working on TPU limitation and crop physiologists working on whole-plant sink limitation. Fabre *et al.* (2019) attempted to link sink limitation at both scales and showed that TPU limitation was more prevalent in panicle-pruned rice plants, especially when grown under 800 μmol mol^−1^ [CO_2_]. The photosynthetic stimulation by elevated [CO_2_] was smaller in pruned plants than in control plants. The dependency of the [CO_2_] response on sink size was also found when comparing five rice genotypes having contrasting panicle/leaf size ratios or sink/source ratios (Fabre *et al.*, 2020). The rate of TPU (*T*_p_), thus *A*_max_ (see Equation 1), declined under sink limitation, increasingly after midday in a diurnal cycle, associated with sucrose accumulation in the flag leaf (Fabre *et al.*, 2019). These findings suggest that TPU limitation to leaf photosynthesis may be regulated by sink feedback at the whole-plant scale. Acevedo-Siaca *et al.* (2021) showed that like *V*_cmax_ and *J*_max_, *T*_p_ estimated for 30 rice accessions had high heritability. Data of Acevedo-Siaca *et al.* (2021) further showed that rice *A*_max_ at CO_2_ saturation was highly heritable but *A*_max_ at ambient [CO_2_] was not, suggesting that genotypic variation in *A*_max_ might be caused by genotypic sink limitation (Fabre and Dingkuhn, 2022).

### Canopy extinction coefficients

The decrease in light incident on leaves at increasing canopy depth commonly follows the Beer–Lambert law:


$$\begin{array}{l} I_{i}=I_{0}e^{-k_{L}L_{i}} \end{array}$$ (4)

where *I*_0_ and *I*_i_ are incoming irradiances at the canopy top and at the layer where the leaf area index (LAI) accrued from the top is *L*_i_, and *k*_L_ is called the canopy light extinction coefficient. The value of *k*_L_ depends somewhat on solar zenith angle, direct versus diffuse light intensity, and canopy size, causing some variation with time of day, cloudiness, and crop development. For a closed canopy under given light conditions, *k*_L_ primarily depends on leaf angle distribution (e.g. Ouyang *et al.*, 2021) and leaf [CHL] (e.g. Gu *et al.*, 2017). Like [CHL] (see above), leaf angle shows significant genetic variation (Li *et al.*, 2015; Truong *et al.*, 2015; Lu *et al.*, 2018). More erect leaves and lower [CHL] allow more light to reach the lower leaf strata, enabling them to contribute more to canopy photosynthesis. In that sense, more prostrate leaves during early growth and erect leaves after canopy closure are beneficial for the whole-season light interception. Likewise, more prostrate leaves at the canopy bottom and more upright leaves at the top allow greater crop light interception. Erect orientation also reduces the risk of photoinhibition in top leaves under high irradiances at noon on sunny days (Horton, 2000; Jaikumar *et al.*, 2021).

There has been recent interest in reducing top-leaf [CHL] to reduce photoinhibition and increase canopy light interception and productivity (Ort *et al.*, 2011; Gu *et al.*, 2017; Walker *et al.*, 2018). However, canopy photosynthesis (*A*_canopy_) depends not only on the light interception achievable with a given amount of photosynthetic resources, but also on how these resources are distributed in the canopy. Mathematical optimization showed that *A*_canopy_ is maximal if these resources are distributed in such a way that the *A*_max_ gradient is comparable with the vertical light profile (Goudriaan, 1995; Sands, 1995). Nitrogen is the most important photosynthetic resource, and leaf nitrogen is observed to decrease with canopy depth (e.g. Evans, 1993), which can be described in analogy to Equation 4:


$$\begin{array}{l} n_{i}=(n_{0}-n_{b})e^{-k_{N}L_{i}}+n_{b} \end{array}$$ (5)

where *n*_0_ and *n*_i_ are nitrogen content of uppermost leaves and of those at the layer *i*, respectively; *n*_b_ is the base leaf nitrogen content, at or below which photosynthesis is nil; and *k*_N_ is the canopy nitrogen extinction coefficient. Assuming a linear increase of *A*_max_ with leaf nitrogen (e.g. Setter *et al.*, 1994; Dreccer *et al.*, 2000; Hikosaka, 2010), the optimization theory predicts that *k*_N_=*k*_L_. However, often the observed *k*_N_ is lower than *k*_L_ (Hikosaka *et al.*, 2016), indicating the possibility of improving *A*_canopy_ via optimizing nitrogen versus light profiles.

Indeed, genotypic variations have been reported in various crop species for *k*_N_, *k*_L_, and their ratio (Dingkuhn *et al.*, 1991; Bertheloot *et al.*, 2008; Sadras *et al.*, 2012; Gu *et al.*, 2017; Zhu *et al.*, 2020; Ouyang *et al.*, 2021). Moreau *et al.* (2012) reported for 16 cultivars that variation is larger for *k*_N_:*k*_L_ than for the *k*_L_ itself, suggesting a potential for optimizing this ratio. Ouyang *et al.* (2021) showed that superior carbon gain between stem-elongating and flowering stages in rice genotypes was mainly explained by a higher *k*_N_:*k*_L_ ratio. However, contrary to the theory, Sadras *et al.* (2012) showed a negative correlation between radiation use efficiency (RUE) and the *k*_N_:*k*_L_ ratio for 13 Australian wheat cultivars released between 1958 and 2007. The negative correlation may result from confounding effects of other traits (e.g. leaf greenness) that also varied with the year of release.

### Stay-green and feedback of nitrogen balance on photosynthesis

Maintaining green surface area longer (stay-green) increases crop photosynthetic duration. There are several types of stay-green (Thomas and Howarth, 2000). Here we focus on stay-green achieved by optimizing nitrogen uptake and allocation to grains. Grain nitrogen comes partly from *de novo* uptake by roots, but also largely from remobilization of nitrogen from vegetative green tissues (Gaju *et al.*, 2014; Shao *et al.*, 2021), especially from photosynthetic enzymes (Mu *et al.*, 2018). Remobilization results in leaf senescence (Sinclair and de Wit, 1976). Without provision of additional nitrogen, grain nitrogen (and thus protein content) generally decreases with yield increases. Such negative relations result from not only dilution by photoassimilates but more from remobilization.

While remobilization is a general process occurring in many crops (Monaghan *et al.*, 2001; Bogard *et al.*, 2010; Wei *et al.*, 2018), genetic differences were observed. Some wheat genotypes are able to accumulate more grain protein than others at the same yield level (Monaghan *et al.*, 2001). This can be achieved by higher post-anthesis nitrogen uptake, according to data on 27 wheat genotypes (Bogard *et al.*, 2010) and 15 doubled haploid wheat lines (Hebbar *et al.*, 2014). Likewise, variation in onset and extent of leaf senescence among nine sorghum genotypes was explained by differences in SLN and post-floral nitrogen uptake (Borrell *et al.*, 2001). Using wheat stay-green mutants, Chapman *et al.* (2021) also showed the link between onset of senescence and grain-filling duration, with an ~14% increase in final grain weight in stay-green genotypes.

There were significant genotypic differences in the amount of nitrogen remobilized from vegetative organs during grain filling among 20 genotypes of wheat (Barraclough *et al.*, 2014). Post-anthesis nitrogen remobilization and the onset of rapid canopy senescence were correlated among 16 wheat cultivars grown in the UK and France (Gaju *et al.*, 2014). Grain demand for nitrogen can also strongly affect source–sink balance during grain filling. Genotypes with higher grain nitrogen concentration tend to be more source limited in rice (Wei *et al.*, 2018), probably due to faster nitrogen remobilization and accelerated leaf senescence. It is unknown if there is any genetic variation in the dynamics of grain nitrogen demand during filling. One can hypothesize that genotypes having lower nitrogen demand in the earlier than in the later grain-filling phase would remobilize less and maintain canopy photosynthesis longer than those that have constant or earlier nitrogen demand.

### Early vigour, partly as a feedforward result of increased photosynthesis

Another way to increase photosynthetic duration is to have an earlier canopy closure, which ensures more interception of light as well as more effective suppression of weeds (Richards, 2000). This can be achieved by increased tillering or branching, or with thinner leaves [higher specific leaf area (SLA)] that would allow faster leaf expansion (Dingkuhn *et al.*, 1999). Selection for high *A*_max_ is not conducive for early vigour if it is achieved at the cost of thicker leaves (Boote and Tollenaar, 1994). Record values of C_3_*A*_max_, >60 μmol m^−2^ s^−1^ at ambient [CO_2_] (e.g. Pearcy and Ehleringer, 1984), were observed for desert plants. Such high values of *A*_max_ associated with thick leaves are not useful for crop plants that require rapid leaf expansion and canopy closure for more light interception during crop establishment.

However, improving leaf photosynthesis without reducing SLA may increase early leaf expansion. In recent studies where leaf photosynthesis was improved by genetic modification (Kromdijk *et al.*, 2016; Driever *et al.*, 2017; Simkin *et al.*, 2017; Shen *et al.*, 2019; South *et al.*, 2019; López-Calcagno *et al.*, 2020; Yoon *et al.*, 2020), improved photosynthesis also resulted in greater leaf area or larger plants that in turn intercept more light. It would be worthwhile assessing to what extent the reported increased biomass was directly caused by higher leaf photosynthetic rates versus indirectly by increased leaf expansion. Using 40 genotypes of ryegrass, Yiotis *et al.* (2021) showed that greater yield gain under elevated [CO_2_] is more likely to occur through exploiting genetic differences in tillering and leaf area rather than in leaf photosynthesis. For different photosynthesis types (among which differences in *A*_max_ are generally large), Atkinson *et al.* (2016) compared 382 grass species. They found that C_4_ species had a 19–88% daily growth advantage over C_3_ grasses at the seedling stage, but this advantage was driven largely by a high SLA (enabling faster leaf expansion), rather than by fast biomass gain per unit leaf area.

In view of the above considerations, SLA should ideally be larger in early growth phases (to accelerate canopy closure) and smaller in later phases (to increase *A*_max_). Domestication and selection seem to have enhanced such SLA dynamics for some crops (rice: Peng *et al.*, 1993), as opposed to others (barley: Yin *et al.*, 1999). Genotypic differences in SLA are significant (Peng *et al.*, 1993; Yin *et al.*, 1999; Zhu *et al.*, 2020). Dingkuhn *et al.* (2001) further showed that the early relative growth rate of rice genotypes was correlated with tillering ability, and SLA was largely responsible for differences in tillering ability and LAI, thereby supporting SLA being a key trait for early vigour.

### Photosynthetic contribution of non-leaf tissues

The contribution of non-leaf tissues to whole-plant photosynthesis and source–sink balance has long been reported (e.g. Biscoe *et al.*, 1975) and was recently reviewed by Tambussi *et al.* (2021). Using the method of covering ears, Maydup *et al.* (2012) estimated that ear photosynthesis contribution to grain filling increased from 10% to 35% among 10 Agentinian wheat varieties released between 1920 and 2008. By designing panicle chambers, Chang *et al.* (2020) measured panicle photosynthetic rates in seven rice cultivars. They represented 20–38% of rates of the corresponding flag leaves. Similarly, Molero and Reynolds (2020) used a custom-made chamber to measure 45 genetically diverse spring wheat genotypes, and showed a variation of 2.8-fold for spike photosynthetic rate. By covering the spikes, they further estimated that the contribution of spike photosynthesis to grain weight was 30–40% in 196 wheat lines. Jiang *et al.* (2006) showed that spike/ear photosynthesis is not only a highly proximal source for grain filling but can also offset the very high local carbon demand for dark respiration. Thus, strategies to increase canopy photosynthesis should consider inflorescence photosynthesis. This is especially relevant if the presence of C_4_-type photosynthesis in developing wheat grains is real (Rangan *et al.*, 2016; contested by Busch and Farquhar, 2016). Significant spike photosynthetic rates also reduce the need to lower panicle height in favour of canopy top-leaf photosynthesis (Setter *et al.*, 1995), as this trait invites humidity-loving pathogens.

## Molecular mapping of photosynthesis-related traits

The natural variations reviewed above represent only phenotypic trait variations. Genetic variations are smaller because of (i) measurement errors; (ii) confounding environmental variation; and (iii) possible differences in nodes of physiological and genetic control. Of the genetic variation, mainly additive effects are utilized in inbred breeding, only dominant alleles can be exploited in hybrid breeding, and complex gene interaction components (epistasis) are difficult to use (Kearsey and Pooni, 1996). To support breeding, it is important to map QTL for traits, providing information on the effect and putative function of loci, and markers as selection tools. This is mostly achieved with bi-parental populations for linkage analysis or association panels for GWAS (genome-wide association study). Here, we review the mapping of the aforementioned photosynthesis-related traits but present them in groups.

### Radiation-use efficiency and crop photosynthesis traits

Yin *et al.* (2003) reported that the dwarfing allele of the major gene *denso* (also designated as *sdw*1) on chromosome (chr.) 3 decreased RUE in a recombinant inbred line (RIL) population of barley. More recently, Molero *et al.* (2019) used GWAS for 150 elite spring wheat genotypes including landraces and synthetically derived lines. They identified 94 single nucleotide polymorphisms (SNP) associated with RUE and biomass at various stages that explained 7–17% of the phenotypic variation. Common SNP markers were identified for grain yield, final biomass, and RUE on chr.5A and chr.7A. Landraces and synthetic derivative lines had higher RUE but lower harvest index (HI), suggesting that RUE has not been improved by breeding. Building on Molero *et al.* (2019), Joynson *et al.* (2021) conducted high-throughput hyperspectral reflectance phenotyping to map wheat photosynthetic capacity, demonstrating that GWAS for photosynthesis traits is feasible in the field (Silva-Perez *et al*., 2020).

Genetic mapping studies mostly focus only on photosynthesis at a specific stage. To study the effects of photosynthesis on crop productivity, Honda *et al.* (2021) phenotyped photosynthetic rate and crop growth rate (CGR) of 76 Koshihikari×Takanari rice chromosome segment substitution lines (CSSLs) during the growing season, and CGR was phenotyped based on biomass sampled at two stages. Cumulative photosynthetic rate during the post-heading phase predicted the CGR during that period well. However, importantly, sustaining high photosynthesis levels was more crucial for CGR than the maximal level, which is usually observed around flowering and followed by a decline. Thus, sustaining high photosynthesis (e.g. via green leaf area duration), rather than maximal rates, is important for increasing CGR and biomass. A genomic region on chr.3 was found to enhance both biomass at harvest and photosynthesis sustenance.

### Leaf photosynthesis and its underlying parameters

Barbour *et al.* (2016) mapped leaf *A*_max_, *g*_s_, and *g*_m_ on 150 doubled-haploid wheat lines, whereby *A*_max_ varied from 22.4 μmol m^−2^ s^−1^ to 35.3 μmol m^−2^ s^−1^, *g*_s_ from 0.50 mol m^−2^ s^−1^ to 1.30 mol m^−2^ s^−1^, and *g*_m_ from 0.27 mol m^−2^ s^−1^ bar^−1^ to 0.94 mol m^−2^ s^−1^ bar^−1^. However, only two QTL were identified for *A*_max_, each explaining 5–7% of phenotypic variation; there were two QTL for *g*_s_, each explaining 5%; and one for *g*_m_, explaining 9% of variation.

Adachi *et al.* (2011a) mapped three QTL for rice flag-leaf *A*_max_ on chr.5, 8, and 11 from a Habataki×Sasanishiki cross and attributed the higher *A*_max_ (Habataki alleles) to higher SLN and *g*_s_. Adachi *et al.* (2011b) confirmed the *A*_max_ allele on chr.8 from Habataki in a Habataki×Koshihikari cross and reported another QTL on chr.4. Each QTL explained 6–9% of *A*_max_ phenotypic variation. The high SLN and *g*_s_ putatively responsible for high *A*_max_ were associated with increased root surface area and hydraulic conductivity, hinting at underlying traits promoting nitrogen uptake. Among backcrossed inbred lines derived from a third cross between Takanari and Koshihikari, two rice lines were identified that had 20–50% higher *A*_max_ than the parental rates (Adachi *et al.*, 2013). In addition to SLN, high *g*_m_ underlined the high *A*_max_ of the lines, due to their higher density and more developed lobes of mesophyll cells. Thus, Tanaka *et al.* (2014) considered SLN, *g*_s_, and *g*_m_ as the main factors for increasing rice *A*_max_, as confirmed by near-isogenic lines for these QTL (Adachi *et al.*, 2014). This differs from the results of Barbour *et al.* (2016) in wheat, where the loci for *A*_max_, *g*_s_, and *g*_m_ were independent. Using CSSLs, Adachi *et al.* (2019) were able to detect several more *A*_max_ QTL, each explaining 8–18% of phenotypic variations. Pyramiding these QTL alleles increased *A*_max_ consistently, and some alleles increased biomass and grain yield. Takai *et al.* (2013) identified the *NAL1* (*Narrow leaf1*) gene underlying one QTL on chr.4. The flowering-date gene *DTH8/Ghd8/LHD1* (Dai *et al.*, 2012) was underlying the *A*_max_-increasing QTL on chr.8 (Adachi *et al.*, 2017).

For rice, another systematic study was conducted involving modelling, using 96 introgression lines. Initially, 1–3 QTL were detected for *A*_max_, *g*_s_, and PSII quantum efficiency, each explaining 4–22% of phenotypic variation (Gu *et al.*, 2012a). The two parents and 11 lines were then selected to measure CO_2_– and light–response curves (Gu *et al.*, 2012b), allowing parameterization of a combined conductance–photosynthesis model of Farquhar *et al.* (1980). Photosynthesis was thus dissected into components *g*_s_, *g*_m_, *V*_cmax_, *J*_max_, κ_2LL_ (conversion efficiency of incident light to electron transport), and *R*_d_ (day respiration). Seven loci significantly affected these model parameters. Each parameter was controlled by 1–3 loci, and most loci controlled several parameters. Assuming additivity, ideotypes were designed, combining positive-effect alleles for the parameters (Gu *et al.*, 2014a). The best combination was projected to improve photosynthesis by ~20% compared with the best of the 13 lines investigated by Gu *et al.* (2012b). Scaled up to crop level by using the crop model GECROS (Yin and van Laar, 2005), a 25% genetic variation in photosynthesis of 25% gave a theoretical increase in biomass of 22–29%. κ_2LL_ was predicted to contribute most to variation in biomass, being more effective than *g*_s_ and *g*_m_ within the range of observed variation.

Among the Farquhar *et al.* (1980) model parameters, *R*_d_ is hard to measure on a large population and is commonly assumed to correlate with leaf respiration in the dark (*R*_dk_). Qu *et al.* (2020) observed *R*_dk_ on 206 rice accessions grown under both indoor and field conditions. *R*_dk_ positively correlated with leaf thickness and [CHL]. GWAS identified an overlapped genomic region on chr.3 for *R*_dk_ in both environments. A single SNP in the promoter region of the *LRK1* (leucine-rich repeat receptor kinase) gene was strongly correlated with the mean annual temperature of the regions where accessions were collected.

### Source–sink traits

The realization that sink capacity co-controls leaf photosynthetic rates via feedback becomes increasingly relevant for improving crop productivity as [CO_2_] rises (Dingkuhn *et al.*, 2020). Because genotypic sink traits strongly affect elevated CO_2_ response (Hasegawa *et al.*, 2013; Fabre *et al.*, 2020), traits well known to breeders will be seen in a new light, such as tiller, phytomere, and floret initiation rates (organogenetic vigour) and inflorescence size.

For reproductive stage sink–source relationships, Wang *et al.* (2020) conducted GWAS for 272 rice accessions, finding 70 QTL influencing 11 traits. Overall, 5–9 QTL were found per trait, each explaining 7–20% of trait phenotypic variation. The *NAL2* (*Narrow leaf2*) gene was found to control a typical sink trait, panicle number per plant, agreeing with *NAL*2 and *NAL*3 encoding the OsWOX3A transcription factor that is broadly involved in organ development (Cho *et al.*, 2013). Another generic mechanism for sink enhancement is T6P-mediated sugar signalling (Dingkuhn *et al.*, 2020). Lyra *et al.* (2021) indicated that beyond proven options for engineered T6P-based sink enhancement, much natural, functionally effective genetic variation in key genes *TPS* and *TPP* exists in wheat waiting to be mined for breeding.

Source–sink trait analyses rarely consider photosynthesis of the inflorescence. Molero *et al.* (2014) identified markers associated with spike photosynthesis contribution to grain yield in a RIL population of wheat. Three QTL were detected that explained 10–24% of the variation in the contribution, highlighting the potential for improving spike photosynthesis.

### Other morpho-physiological traits

Canopy photosynthesis (*A*_canopy_) is a complex trait integrating many physiological and morphological components. Leaf angle is particularly important and has been a pivotal trait for Green Revolution breeding. The role of the rice semi-dwarfing (*SD*1) gene in reducing height, changing leaf angle, and increasing tillering is history and requires no review here. In sorghum, Truong *et al.* (2015) identified 2–4 loci, explaining 12–38% of phenotypic variation in leaf angle in each of two RIL populations. Alleles of the gene *dwarf-3* were shown to change leaf inclination by up to 34°. Li *et al.* (2015) phenotyped three connected RIL maize populations (538 RILs) for leaf angle. Seventeen identified QTL together explained ~60% of phenotypic variance. Also for maize, Lu *et al.* (2018) conducted GWAS with 80 inbred lines. Twenty-two SNPs were detected for leaf angle, with five each explaining 5–22% of the phenotypic variation.

Stay-green can maintain high *A*_canopy_ during grain filling. To map stay-green, Chapman *et al.* (2021) developed RIL populations segregating for the timing of senescence in wheat. They found two independent loci of 4.8 Mb and 16.7 Mb in size encompassing 56 and 142 genes. Combining association analysis with variant effect prediction, they identified effective SNPs in the locus of *NAM-1*, a gene associated with grain protein content. This provides a molecular basis for the earlier discussed ‘nitrogen remobilization’.

Stay-green also depends on leaf [CHL], which in turn determines leaf photon absorptance and affects canopy *k*_L_. Wang *et al.* (2015) conducted GWAS for [CHL] using a collection of 529 diverse rice accessions. A total of 46 loci were identified. Three F_2_ mapping populations with parents selected from the panel were developed to validate the major GWAS signals, each providing 1–2 QTL that explained 10–20% of phenotypic variation. *Ghd7* (*Grain number, plant height, and heading date7*), being a major regulator of nitrogen uptake (Wang *et al.*, 2021), was a major underlying gene for [CHL] at heading stage. Enhanced expression of *Ghd7* decreased [CHL]. On another locus, *NAL1—*the gene involved in cell division and auxin-mediated expansion—was identified (Lin *et al.*, 2019).

For early vigour, Yin *et al.* (1999) reported several QTL for early-stage SLA in a barley RIL population, together explaining >40% of phenotypic variation. Zhang *et al.* (2017) phenotyped four traits including seedling shoot length in 132 rice RILs. They detected 10–28 QTL, each explaining up to 14% of phenotypic variation. Chen *et al.* (2019) used 744 rice accessions to detect QTL for tiller number, plant height, and above-ground dry weight at the seedling stage, detecting 42 QTL.

## Outlook

Our review shows large phenotypic variations (sometimes >2-fold) for steady-state photosynthesis traits. There are few QTL identified for photosynthesis traits *per se* such as *A*_max_, and these QTL accounted for a low percentage of phenotypic variations, typically <20%. In contrast, more QTL were reported for sink size (that feeds back on photosynthesis) or morpho-physiological traits (that affect canopy productivity and duration), together explaining a much higher percentage of their phenotypic variation (typically >60%).

Measurement error (Gu *et al.*, 2012a) and the uncertainty in innate reasons for observed variability of photosynthesis (Fabre and Dingkuhn, 2022) might explain why its variation remains poorly explained. However, this does not mean that there is little opportunity to improve photosynthesis. Instead, breeding may have selected photosynthesis-related traits that probably partly contributed to recent yield progress (Fischer *et al.*, 2014). Our review showed that apart from some existing variation in the photosynthetic apparatus itself, much of the molecular basis of photosynthesis QTL resides in genes controlling nitrogen use, source–sink relations, leaf morphology, or senescence patterns. We thus hypothesized that some genotypic variation in *A*_max_ (and its degree of heritability) might actually be due to variation in sink limitation. In addition, traits for both photosynthetic rate and its sustenance during grain filling are strongly related to nitrogen-related traits.

Given these considerations, we conducted an analysis using the crop model GECROS to evaluate how improving photosynthesis can enhance crop productivity (Box 2; Table 1). The model confirmed that the maximum benefit can be achieved from simultaneous improvement of other traits; in particular, proportionally increased root nitrogen uptake is required to significantly improve productivity. The model also showed that understudied electron transport parameters were much more effective than the commonly studied *A*_max_. This is because photosynthesis of most leaves at most hours in a canopy, even on sunny days, are light subsaturated (electron transport limited). This is corroborated by observations of Qu *et al.* (2017) and Taniyoshi *et al.* (2020) on the importance of *A*_low_ and supports views on optimizing photosynthetic light reactions (Yin and Struik, 2021; Walter and Kromdijk, 2022). Thus, improving photosynthesis is not merely a matter of increasing *A*_max_ but should improve multiple parameters synergistically, allowing for high canopy photosynthesis and duration.

**Table 1.** Simulated advantage (%) in radiation use efficiency (RUE) and above-ground biomass (31 year average) as a result of improving individual traits or trait combinations over the baseline simulation

Trait type	Parameter^*a*^	Parameter values		Advantage over the ­baseline (%)^*b*^	
		Baseline	Improved	RUE	Biomass
Photosynthetic	1 χ_Vcmax_	75	90	0.2	0.0
	2 χ_Jmax_	100	120	3.7	5.0
	3 Φ_2LL_	0.78	0.85	2.8	3.0
	4 *g*_s_	Variable	1.2×baseline	0.8	1.0
	5 χ_gm_	0.125	0.150	0.8	1.0
	6 TPU limitation	Present	Removed^*c*^	1.1	1.3
Morpho-physiological	7 Leaf angle	65	52	-0.3	0.0
	8 *k*_N_:*k*_L_	0.80	0.96	2.4	2.5
	9 Stay-green^*d*^	Baseline	Improved	1.6	2.1
	10 SLA	0.030	0.036	–1.9	–1.8
	11 Non-leaf tissue^*e*^	Baseline	Improved	2.8	3.1
Nitrogen uptake^*f*^	12 *N*_umax_	20	24	10.7	14.6
Trait combination	Photosynthetic traits 2–6			14.0	13.0
	Morpho-physiological traits 8, 9, and 11			6.9	6.7
	Traits 2–6, 8, 9, and 11			21.9	19.1
	Traits 2–6, plus 12 (i.e. *N*_umax_)			29.0	31.5
	Traits 8, 9, 11, plus 12 (i.e. *N*_umax_)			18.9	22.6
	Traits 2–6, 8, 9, 11, plus 12 (i.e. *N*_umax_)			37.2	39.1

^*a*^ Parameter definition: (1) χ_Vcmax_, slope of *V*_cmax_ (maximum rate of carboxylation by Rubisco) versus leaf nitrogen (μmol g^−1^ N s^−1^); (2) χ_Jmax_, slope of *J*_max_ (maximum rate of photosynthetic electron transport) versus leaf nitrogen (μmol g^−1^ N s^−1^); (3) Φ_2LL_, PSII electron transport efficiency under limiting light (mol mol^−1^); (4) *g*_s_, stomatal conductance (which is variable, depending on light, CO_2_, temperature, and vapour pressure); (5) χ_gm,_ slope of *g*_m_ (mesophyll conductance) versus leaf nitrogen (mol g^−1^ N s^−1^ bar^−1^); (6) TPU (triose phosphate utilization)-limited photosynthetic rate, set in its simplest form as 3*T*_p_−*R*_d_ (Sharkey, 1985), which can be derived from Equation 1 with α_S_=0; where *R*_d_ is day respiration, and *T*_p_ is the rate of TPU with χ_*T*p_ (slope of *T*_p_ versus leaf nitrogen) being 5 μmol g^−1^ N s^−1^ (Harley *et al.*, 1992); (7) leaf angle from the horizontal line at the early phase (°); (8) the leaf nitrogen to light extinction coefficient ratio (–); (9) stay-green coefficients in relation to grain demand for nitrogen (–); (10) specific leaf area (SLA) at the early phase (m^2^ g^−1^); (11) coefficients for quantifying the photosynthesis contribution from non-leaf tissues (–); (12) season-long crop nitrogen uptake (g N m^−2^).

^*b*^ Simulated 31 year average using the baseline parameter values (taken from Yin and Struik, 2017) was 2.57 g (MJ PAR)^–1^ for RUE and 19.6 t ha^–1^ for above-ground biomass.

^*c*^ The removal of this TPU limitation was simply assumed to be the ‘improved’ form because of the lack of understanding of the whole-plant physiology to fully represent the extent of sink feedbacks on source.

^*d*^ Stay-green traits are modelled in GECROS in relation to nitrogen remobilization from vegetative organs in support of grain filling. Therefore, parameters were changed by 20% to allow slower remobilization, thereby, improving stay-green status.

^*e*^ In the GECROS model, green surface area index (GAI) includes leaf and green non-leaf tissue areas. Here, parameters were changed by 20% to allow more non-leaf tissue areas.

^*f*^ Nitrogen uptake (*N*_umax_) is not an input parameter but a simulated output in the default GECROS model. Here we set *N*_umax_ as a controlled crop variable, so as to separate the impact of improving photosynthetic or morpho-physiological traits from that of increasing nitrogen uptake on crop productivity (see Box 2). For this simulation, the dynamics of crop N uptake were assumed to follow a sigmoid pattern, with *N*_umax_ (default value=20 g N m^–2^) as the total N uptake during the growing season (Setter *et al.*, 1994). A 20% increase scenario was to increase *N*_umax_ by 20% but with the uptake proportion for each specific day unaltered.

**Box 2. Modelling potential RUE and biomass gains from pyramiding photosynthetic traits**

We used the crop model GECROS (Yin and van Laar, 2005; Yin and Struik, 2017) to assess the potential of improving various traits for increasing crop productivity. The model version here was used by Yin and Struik (2017) and Kadam *et al.* (2019) but incorporates a multilayer module for computing canopy photosynthesis. We first assess the traits individually and then assess them in combination (Table 1). We used weather data of 1980–2010 at the International Rice Research Institute (IRRI), Philippines, for simulation. Baseline GECROS parameter values were taken from Yin and Struik (2017), predicting 31 year average above-ground biomass at 19.6 t ha^–1^, representing the best check rice cultivar observed at IRRI where nitrogen (N) uptake (*N*_umax_) is ~20 g N m^−2^ for well-managed crops (Setter *et al.*, 1994). Considering typical phenotypic variations and percentages explained by identified QTL as reviewed in the main text, we assumed that each trait (model parameter) can be improved in a favourable direction by 20% (except Φ_2LL_ whose maximum value is ~0.85, Björkman and Demmig, 1987) given that it is unknown how much a trait can be improved by breeding.

Modelled impact of trait improvement was mostly similar on RUE and biomass, but not identical due to pleiotropic effects simulated on light interception. Among six photosynthetic traits (*V*_cmax_, *J*_max_, Φ_2LL_, *g*_s_, *g*_m_, and TPU limitation removal), increasing *V*_cmax_ by 20% did not increase biomass. In contrast, improving electron transport parameters, *J*_max_ (+20%) and Φ_2LL_ (+9%), had the greatest impact and increased biomass by 5% and 3%, respectively (Table 1). Improving CO_2_ diffusion parameters (*g*_s_ and *g*_m_) by 20% and removing TPU limitation each increased biomass by ~1%. The greater importance of improving *J*_max_ versus *V*_cmax_ was reported previously (Gu *et al.*, 2014a; Yin and Struik, 2015), suggesting a possible overinvestment of N in Rubisco in existing cultivars. Improving *V*_cmax_ or removing TPU limitation only increases light-saturated *A*_max_ (see Equation 1), which is relevant for top leaves around noon on sunny days. In contrast, improving *J*_max_ not only increases *A*_max_ but also lifts up the entire light–response curve of electron transport, thus also increasing light subsaturated rates. This, especially if combined with improved Φ_2LL_, has a significant consequence on productivity of most leaves at any time of day and season. This is supported by Qu *et al.* (2017), reporting that genotypic variation in productivity was associated with *A*_low_ (photosynthesis under low light). Although a limited scope for improving Φ_CO2_ was previously suggested (Austin, 1989), there is evidence for large variation in chloroplast electron transport, with 4-fold differences reported for barley (Burkey, 1994). Furthermore, with *J*_max_, Φ_2LL_, *g*_s_, and *g*_m_ all improved and TPU limitation removed, combined effects were higher than the sum of the individual effects, increasing RUE and biomass by ~14% (Table 1).

In addition to the sink limitation at a biochemical level (the TPU limitation), there are feedback effects of morpho-physiological sinks (such as grain number and size) on photosynthesis (see the main text). However, further research is needed to model this feedback. Among five modelled canopy morpho-physiological traits, adjusting leaf angle and SLA for early vigour had no or slightly negative effects on biomass (Table 1). Improving *k*_N_:*k*_L_, stay-green by adjusting N remobilization, and improving non-leaf photosynthesis each increased biomass by 2–3%. Combined improvement of the latter three traits increased biomass by 6.7% (Table 1), slightly less than the sum of individual effects. Overall, improving these morpho-physiological traits had a smaller effect, compared with the combined improvement of photosynthetic parameters. Thus, these morpho-physiological traits are probably near optimal due to past breeding. Nevertheless, effects of combining photosynthetic and morpho-physiological traits seemed additive, together increasing biomass by ~19% (Table 1).

Improving photosynthetic traits may be combined with increasing N uptake. Higher leaf N content enables higher photosynthetic rates and can be associated with greater root surface area and conductivity (Hikosaka, 2010; Adachi *et al.*, 2011b). The causality between improved photosynthesis and root N uptake ability is unclear. Increasing *N*_umax_ by 20% increased simulated RUE by 10.7% and biomass by 14.6% (Table 1), in line with the importance of past breeding for more N input-responsive cultivars (Sinclair *et al.*, 2019). Increasing N uptake combined with either improved photosynthetic or morpho-physiological traits resulted in synergistic effects. When all these traits were improved, RUE and biomass increased by ~37% and 39%, respectively (Table 1). These values are similar to the projected impact of introducing the full crop C_4_ mechanism into rice (Yin and Struik, 2017).

Many mapping studies use diversity panels showing very large phenotypic trait variation. For breeding to improve yield, only the portions of variation beyond best-performing cultivars are useful. Our modelling suggests that many structural crop traits (such as stay-green or early vigour) have already been largely optimized by breeding, with limited scope for further improvement (Box 2), at least under current ambient [CO_2_]. However, natural variation in photosynthesis remains largely unexploited (Driever *et al.*, 2014; Gu *et al.* 2014b; Faralli and Lawson, 2020). In fact, as shown in this review, there are cases where dwarfing seems to have decreased photosynthesis or RUE (e.g. Yin *et al.*, 2003; Molero *et al.*, 2019; Mathan *et al.*, 2021). Thus, our view is somewhere between that of photosynthesis biologists and crop physiologists: improving the photosynthetic apparatus can contribute to yield improvement, namely regarding its electron transport components; but enabling traits such as root nutrient uptake and sink capacity must be co-selected by breeding. As stated in Box 2, the synergistic impact of exploiting natural variation of multiple components could match the impact of implementing the full mechanism of maize C_4_ photosynthesis in C_3_ crops.

We discussed little about photosynthesis under fluctuating light conditions. Acevedo-Siaca *et al*. (2020a, b) showed that genotypic variation in non-steady-state rice photosynthesis did not correlate with that under steady-state conditions, but exceeded it. However, Salter *et al.* (2020) identified a QTL for Rubisco activation rate under fluctuating light overlapping with a QTL for steady-state photosynthesis in barley. Taniyoshi *et al.* (2020) showed that the cumulative CO_2_ fixation rate during the 10 min after the transition from low to high irradiance was not correlated with the rate at the high irradiance but significantly correlated with the rate at low light. The potential significance of such traits for crop improvement requires further study.
